# Beta bursts during continuous movements accompany the velocity decrement in Parkinson's disease patients

**DOI:** 10.1016/j.nbd.2019.03.013

**Published:** 2019-07

**Authors:** Roxanne Lofredi, Huiling Tan, Wolf-Julian Neumann, Chien-Hung Yeh, Gerd-Helge Schneider, Andrea A. Kühn, Peter Brown

**Affiliations:** a Nuffield Department of Clinical Neurosciences, University of Oxford, Oxford, UK; b Medical Research Council Brain Network Dynamics Unit, University of Oxford, Oxford, UK; c Movement Disorders and Neuromodulation Unit, Department of Neurology, Charité – Universitätsmedizin Berlin, Berlin, Germany; d Berlin Institute of Health (BIH), 10178 Berlin, Germany; e Department of Neurosurgery, Charité – Universitätsmedizin Berlin, Berlin, Germany

**Keywords:** Parkinson's disease, Bradykinesia, Subthalamic nucleus, Beta oscillations, Beta bursts

## Abstract

Bradykinesia is reported to correlate with subthalamic beta power (13–35 Hz) recorded at rest in Parkinson's disease (PD). Pilot studies suggest adaptive deep brain stimulation triggered by amplitude threshold crossings of beta activity defined at rest is effective. This is puzzling, given that beta is suppressed during repetitive movements when bradykinesia becomes apparent. Recently, increased beta power in PD has been linked to beta bursts. Here we investigate whether beta bursts also occur during repetitive movements and relate to progressive decrement in movement velocity. Therefore, subthalamic local field potentials were recorded in 12 PD patients off medication while performing 30s blocks of rotatory movements alternating with rest periods. Bursts were defined separately for the low (13–20 Hz) and high (20–35 Hz) beta band using thresholds defined at rest. As expected, velocity significantly decreased within movement blocks. Despite the sustained suppression of both beta sub-bands, bursts could still be detected during movement. Beta bursts were reduced in amplitude, duration and rate during movement with beta rate correlating best with beta power. A mixed-effects linear model revealed that percentage time spent in beta bursts predicted velocity decreases better than averaged power. This correlation was specific for the low beta band. Our results link beta bursts during movement to bradykinesia. This helps explain how beta activity may contribute to bradykinetic movement decrement even though mean beta power is reduced during movement. Moreover, our findings help explain the effectiveness of adaptive DBS triggered off beta bursts, even though these may be defined with respect to beta levels at rest.

## Highlights

• Beta bursts as defined at rest occur during ongoing movements in the subthalamic nucleus of Parkinson's disease patients.
• Movement associated beta power decrease involves a reduced rate and amplitude of beta bursts when compared to rest.
• Beta bursts during movement may contribute to the pathognomic decrease of velocity in Parkinson's disease.

## 1 Introduction

Parkinson's disease (PD) is a common neurological disorder with bradykinesia as its core motor symptom. Bradykinesia describes the slowness of movement initiation and the progressive decrement in movement velocity and amplitude during repetitive movements that is specific for PD. (Rodrigues et al., 2009; Ling et al., 2012; Rocco et al., n.d.) It is particularly well controlled by chronic deep brain stimulation (DBS), an efficient treatment option for PD patients in whom dopaminergic medications no longer provide consistent benefit (Deuschl et al., 2006; Schuepbach et al., 2013). Recordings from externalized DBS-electrodes have revealed a correlation between the severity of bradykinesia and the extent of averaged beta amplitude (13–35 Hz) in the subthalamic nucleus (STN) (Kühn et al., 2006; Neumann et al., 2016; Oswal et al., 2016). Subthalamic beta power is reduced in parallel with symptom alleviation by both dopaminergic medication (Kühn et al., 2006; Kühn et al., 2009) and DBS (Oswal et al., 2016; Eusebio et al., 2011; Kuhn et al., 2008). Accordingly, beta activity has been considered a neurophysiological correlate of motor impairment and has been used as local feedback parameter for adaptive instead of continuously delivered DBS. There are several approaches to adaptive DBS. One uses a STN beta amplitude threshold to turn stimulation on and off and has proven to have a similar or better efficacy / side-effect profile than conventional DBS (Little et al., 2016a; Little et al., 2013; Little et al., 2016b; Pina-Fuentes et al., 2017). The beneficial effects of this form of adaptive DBS and of dopaminergic medication have recently been linked to a shortening of pathologically prolonged and elevated beta episodes, so called beta bursts (Tinkhauser et al., 2017a; Tinkhauser et al., 2017b). This is in line with findings from physiological brain activity, where beta bursts preceding a movement have been associated with a slowing of subsequent movements (Leventhal et al., 2012; Shin et al., 2017). However, it has not been reported yet whether beta bursts are also present during continuous movements and if so, what effects their occurrence might have on movement velocity. Rather, the current understanding derived from non-invasive and intracerebral recordings in healthy primates and humans, as well as PD patients, is that continuously performed non-isometric movements are accompanied by a sustained suppression of beta power below its level at rest (Androulidakis et al., 2008; Joundi et al., 2013; Muthukumaraswamy, 2010; Steiner et al., 2017; Kühn et al., 2008; Erbil & Ungan, 2007; Cassim et al., 2000; Bichsel et al., 2018). Yet, bursts of increased beta power may not have been captured in previous studies, as beta power was routinely averaged across the time domain. Here we hypothesize that subthalamic beta bursts also occur during repetitive movements in PD patients and may relate to the progressive decrement of velocity in bradykinesia termed the *sequence effect*. Our findings help explain two clinico-physiological paradoxes; how may beta activity contribute to the sequence effect and how can adaptive deep brain stimulation triggered off beta bursts improve bradykinesia when mean beta power is reduced during movement (Johnson et al., 2016)?

## 2 Methods and materials

### 2.1 Patients and surgery

12 subjects with idiopathic Parkinson's disease (mean disease duration 10.5 years, range 5–18 years; mean age 62.8 years, range 47–72 years; four women; further clinical details given in Table 1) undergoing stereotactic functional neurosurgery for bilateral implantation of DBS electrodes in the STN were enrolled in the study. All subjects provided written informed consent which was approved by the local review boards of the Charité - Universitätsmedizin Berlin and in accordance with the standards set by the Declaration of Helsinki. DBS electrode extension cables were externalized in a brief postoperative interval of 5–7 days for clinical testing, allowing the recording of local field potentials (LFP) from the STN. In 9 subjects, the permanent quadripolar macroelectrode used was model 3389 (Medtronic Neurological Division, Minneapolis, MN, USA) with four platinum‑iridium cylindrical surfaces (1.27 mm diameter, 1.5 mm length) and a centre-to-centre separation of 2 mm. In 3 subjects, directional leads (Boston Scientific, Marlborough, MA) with 2 cylindrical (most ventral and most dorsal contact, here termed contacts 1 and 8) and 2*3 segmented surfaces (1.5 mm^2^) with a centre-to-centre separation of 2 mm (here termed contacts 2, 3 and 4 for the ventral segmented ring and 5, 6 and 7 for the dorsal segmented ring) were implanted. In all subjects, correct DBS-electrode placement was confirmed by intraoperative microelectrode recordings and test stimulation. In 10/12 subjects, post-operative CT imaging was available (case 2 and 12 did not have postoperative images in our centre) and used for localization of DBS-electrodes following the semi-automatic approach implemented in the Lead-DBS toolbox (Horn et al., 2018; Horn & Kühn, 2015). In brief, preoperative T2 and T1 weighted MR images were coregistered to postoperative CT scans and normalized to MNI 2009b NLIN Asym standard space. DBS contact artefacts in CT scans were visualized and marked to obtain 3D coordinates in MNI space of all contacts for all available scans.

**Table 1:** Clinical details.

Case	Age/Gender	Disease duration (years)	Symptoms	Handedness	More affected side	UPDRS ON/OFF Medication	Brady-kinesia Subscore^a^
1	62/M	15	Tremor, Bradykinesia	Right	Right	21/24	4
2	66/M	8	Bradykinesia, Rigidity	Right	Right	17/26	5
3	72/M	9	Tremor, Bradykinesia, Rigidity	Right	Left	20/35	5
4	63/M	16	Bradykinesia, Rigidity	Right	Left	29/42	6
5	72/F	5	Bradykinesia, Rigidity	Right	Right	19/34	7
6	61/F	10	Tremor, Bradykinesia, Rigidity	Right	Right	4/20	4
7	47/F	7	Tremor, Bradykinesia, Rigidity	Right	Left	6/29	6
8	58/F	7	Tremor, Bradykinesia	Right	Right	10/32	5
9	57/M	18	Tremor, Bradykinesia	Right	Left	20/41	4
10	65/M	12	Bradykinesia, Rigidity	Right	Left	39/55	6
11	69/M	9	Tremor, Bradykinesia	Right	Right	23/33	6
12	62/M	10	Bradykinesia, Rigidity	Right	Right	30/47	6

a Sum of bradykinesia sub-items in MDS-UPDRS-III of more affected upper limb (finger taps, hand movements, supination/pronation movements) in the OFF medication state.

### 2.2 Paradigm and recordings

**Fig. 1:**
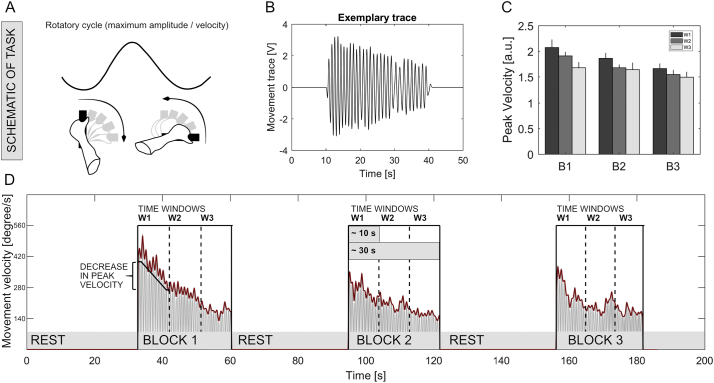
Schematic of task and examplary behavioural results. (A) Subjects were asked to continuously rotate a swivelling handle as quickly and with the largest amplitude possible for a block of 30 s (rotatory cycle in black). (B) Example movement trace from first movement block in case 1. (C) Each subject performed three blocks (B1–3) and velocity dropped across the blocks and also within the blocks which were each divided into three windows. Data averaged across subjects are shown. (D) Shown is an example movement trace from case 5. The movement velocity is shown in light grey. Peaks of movement velocity were interpolated (red trace), resulting in a continuous estimation of peak velocity in each 10s window (W1–3) of each block. Patients had 30 s rest between each block. To determine the change in velocity within each window we took the difference between the mean interpolated peak velocity over the 1 s at the beginning and end of each window. This is shown schematically for the first window of the first block in the figure. There was a stepwise decrease of movement velocity when averaging across movement blocks and across windows within a block. (For interpretation of the references to colour in this figure legend, the reader is referred to the web version of this article.)

LFP-recordings were performed after the subjects had been withdrawn from dopaminergic medication for at least 12 h (OFF state). Subjects were comfortably seated in an armchair and asked to continuously rotate a swivelling handle as quickly and with the largest amplitude as possible for 30 s with their clinically more affected upper limb (right: *n* = 7, left: *n* = 5). Every movement block was preceded and followed by 30 s of rest recording. This sequence was repeated with the same instructions three times (see Fig. 1). Subjects could initiate and execute the repetitive movement in their own time so that it was self-paced in nature. LFPs were recorded between adjacent contact pairs in the 9 subjects with the 3389 electrode model. In the 3 cases with directional leads, bipolar recordings were obtained by referencing each contact to the lowermost contact of the electrode. The recordings were then re-referenced offline to approximate the bipolar recordings derived from adjacent pairs of the circular contacts of the 3389 electrode model (for example: (STNR12 + STNR13 + STNR14)-(STNR15 + STNR16 + STNR17)). Signals were amplified (x 50.000) and low (1 kHz) and high pass (0.5 Hz) filtered using a D360 amplifier (Digitimer Ltd., Welwyn Garden City, Hertfordshire, UK). Data were sampled at 2 kHz on the analog to digital converter (1401). All data were low-pass filtered below 500 Hz and down sampled to 1 kHz offline for further analysis. 9/12 patients also performed the same task several months after implantation of an impulse generator with the option of recording local field potentials where the signal-to-noise ratio allowed only a more limited analysis of the dynamics of beta activity (Steiner et al., 2017).

### 2.3 Data analysis and signal processing

**Supplementary Fig. 1:**
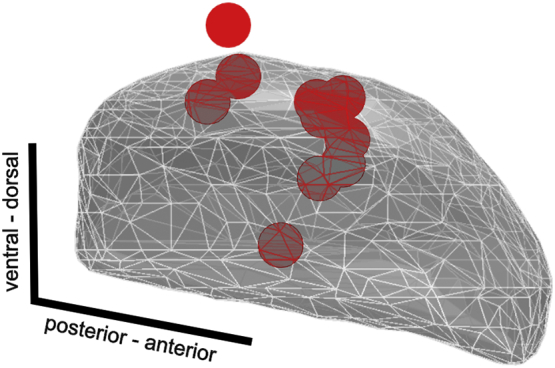
Localization of highest beta power.

Analyses of both behavioural and electrophysiological data were performed in MATLAB (version R2016a; The MathWorks, Natick, USA) using custom Matlab code based on the Statistical Parametric Mapping (Litvak et al., 2011a) and Fieldtrip (Oostenveld et al., 2011) toolboxes. Segments with visually detected artefacts were removed before recordings were down-sampled to 200 Hz and high-pass (3 Hz) and notch filtered (48–52 Hz) to limit movement artefacts and effects of line noise. During movement, the local maxima of the movement velocity (first derivative of movement trace) were automatically detected (matlab function: findpeaks, minimal peak height 0.01, minimal peak distance 30 data points). All automatically defined time points were visually checked and adjusted if necessary. By interpolating between the peaks of movement velocity, a continuous measurement of movement velocity across movement time was obtained which mitigated the effects of directional changes in movement. The interpolated traces were smoothed with a moving average Gaussian smoothing kernel of 50 ms. LFP recordings were transferred to the frequency domain using Morlet wavelets with 10 cycles and a frequency resolution of 1 Hz. Power-spectra were normalized to the sum of total power of 5–45 Hz and 55–95 Hz over the entire recording session to allow comparison across subjects. The bipolar channel with highest peak in the beta band (13–35 Hz) during rest, contralateral to the moved hand, was selected for further analyses. This selection was based on previous reports that demonstrated a significant correlation between contact-pair location in the sensorimotor part of the STN and beta power (Horn et al., 2017). In the present cohort, post-hoc verification of contact pair localization, defined as the Euclidean midpoint between adjacent contacts (Horn et al., 2017; Neumann et al., 2017), confirmed that highest beta power coincided with location in the sensorimotor STN, see Supplementary Fig. 1. Moreover, selected contact pairs were on average 3 ± 0.29 mm distant to a previously reported optimal target location for best clinical DBS outcome in PD. (Caire et al., 2013) The wavelet amplitude was separately averaged across the low beta (13–20 Hz) and high beta (20–35 Hz) sub-bands as distinct roles for both sub-bands in motor control and with respect to dopamine-responsivity have been reported (Oswal et al., 2016). Each power amplitude trace was z-scored (*X-μ/δ*) over the entire recording session and smoothed with a moving average Gaussian smoothing kernel of 175 ms. A burst was defined separately for the high and low beta sub-bands when the instantaneous normalized power exceeded the 75th percentile of the signal amplitude distribution across the rest periods of the recording in the respective frequency band (Tinkhauser et al., 2017a). We took the 75th percentile to define bursts as this has been the one used in previous studies of PD patients in the literature (Tinkhauser et al., 2017a; Tinkhauser et al., 2017b; Tinkhauser et al., 2018; Torrecillos et al., 2018; Lofredi et al., 2018a; Lofredi et al., 2018b). Importantly, using this threshold it has been shown elsewhere that bursts in the STN LFP overlap bursts in the ipsilateral EEG and contralateral STN LFP more often than predicted by chance from permuted data. Moreover, the phase synchronization between cerebral cortex and STN is greater during STN beta bursts than outside of these bursts (Tinkhauser et al., 2018). This is evidence that bursting tends to be synchronised across the basal-ganglia cortical circuit and is not simply the product of a thresholded, time-varying local signal. In line with this, we have previously shown that the pattern of findings is similar regardless of whether the 55th to 90th percentile thresholds is used to define bursts (Tinkhauser et al., 2017a). Another important reason for using the 75th percentile to define bursts is that it equates to the median threshold used in amplitude-responsive adaptive DBS when allowance is made for the processing and ramping applied during adaptive DBS (Little et al., 2016a; Little et al., 2013). Thus the findings with this threshold are relevant to the delivery of adaptive DBS (Tinkhauser et al., 2017a). However, to show that the correlation between change in velocity and low beta bursting is similar regardless of the exact threshold applied, we also tested additional linear mixed-effects models with results derived from thresholding with the 55th, 65th, 85th and 95th percentile. Beta burst duration was defined as the time spent over the predefined threshold. Threshold crossings lasting shorter than 100 ms were not considered, so that bursts involved more than one complete oscillation cycle. The amplitude of a beta burst was defined as the area under the curve between signal and threshold line. Beta burst properties at rest and during movement were compared separately for both beta sub-bands. The distribution of burst durations was considered by categorizing them into five time windows of 100 ms starting from 200 ms to >600 ms in duration.

### 2.4 Statistics

**Fig. 2:**
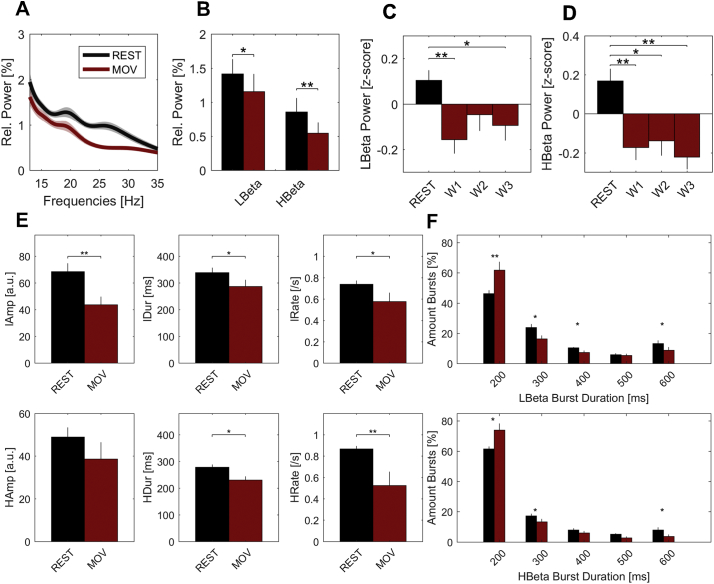
Averaged power spectra and burst properties at rest and during movement. (A) Group average power spectra were estimated separately over movement (red) and rest periods (black). (B) When averaging over the low (13–20 Hz) and high (20–35 Hz) beta sub-bands, there was a significant power decrease in both beta sub-bands during movement. (C, D) Both low and high beta power were continuously suppressed when averaged over 10 s windows within movement blocks. (E) Group averaged data. Beta burst properties during movement (red bars) and rest (black bars) periods were compared separately for the low (upper panel) and high beta sub-bands (lower panel). There was a significant decrease in burst duration and rate during movement when compared to rest, in both beta sub-bands. Additionally, burst amplitude during movement was significantly decreased in the low beta band when compared to rest. (F) During movement, the percentage amount of short bursts (<200 ms) was higher. At rest, the amount of longer bursts was higher. Shaded areas in A and error lines indicate standard errors of the mean. Means and standard errors of the mean are shown. *P < .05; **P < .01. (For interpretation of the references to colour in this figure legend, the reader is referred to the web version of this article.)

Non-parametric Monte Carlo permutation tests were used for statistical analyses. Permutation tests do not rely on assumptions about the underlying data distribution. Note that the interchanged values always stem from the same physiological source and differ only in the test condition in which they occur. To illustrate the method we refer to the comparison between high beta power at rest and during movement, as shown in the right bar plots in Fig. 2B. For legibility, high beta is abbreviated beta in the following. First, beta power was averaged separately over rest and over movement within each patient. Thereby, two groups of twelve beta values at rest and twelve corresponding beta values during movement were generated. We refer to this distribution as the original distribution. We then averaged the beta power at rest across subjects and the beta power during movement across subjects and subtracted mean beta power at rest from mean beta power during movement. In this specific case the mean difference between beta power during movement and at rest was −0.3118, showing that beta power at rest was higher than beta power during movement in the original distribution. In a second step, we created a shuffled distribution by randomly interchanging beta values averaged in each subject over rest and beta values averaged in each subject during movement to give a shuffled distribution. We then averaged the shuffled beta power at rest across subjects and the shuffled beta power during movement across subjects and subtracted mean beta power at rest from mean beta power during movement in the shuffled distribution. The shuffling procedure was randomly repeated 5000 times to generate 5000 mean difference estimates. The mean difference between beta power at rest and beta power during movement in our original distribution was then compared to that in the distribution of 5000 mean differences generated from the shuffled data. If the mean difference in the original data was outside the 95% confidence limits of the mean difference of the shuffled data then this was considered a significant difference.

Rank-based Spearman correlations were calculated if data deviated significantly from a normal distribution as assessed by Kolmogorov-Smirnov tests. Otherwise, linear Pearson correlations were conducted. Correlation coefficients were Fisher transformed before averaging and then back-transformed. Results are reported as mean ± standard deviation and considered significant at an α-level of 0.05 after correction for multiple comparisons by controlling for the false discovery rate (FDR) (Benjamini & Hochberg, 1995). Averaged LFP power and percentage time with burst activity were derived from the activity over 3 windows per 30-s movement block (~10 s per window; see Fig. 1B, D). To assess the sequence effect, the difference in velocity averaged over 1 s (corresponds to approximately two rotations) at the beginning and the end of each time window (~10 s) was calculated. In addition, we repeated analyses when averaging across windows of ~5 s to test for the consistency of any identified effects. Separate linear mixed effects regression models were compared to assess the relationship between change in velocity and mean power or bursting in both beta sub-bands using the Matlab function *fitlme*. Linear mixed effects regression models have excellent statistical power, as they allow for both fixed and random effects as well as for consideration of non-independence in the data, which arises from a hierarchical structure (here the consideration of both individual subject data and cross-subject results). In our implementation, the change in velocity was set as the dependent variable. Averaged power, change in averaged power or percentage time spent in bursting were entered into the model as fixed effects, separately for the low or high beta sub-band. In order to take into account the cross-subject variability in the linear regression intercepts and slopes between the dependent variable and the considered independent variables, the intercepts and the slopes were considered as additional random factors grouped by subjects. Thus, the fixed effects give the estimated population mean values of the slopes in the tested within-subject relationship across all subjects. To control for effects of non-normal distribution, significant models were compared to results after transforming all model inputs to normal distribution (van Albada & Robinson, 2007). Window duration was kept relatively long (10 or 5 s) to observe the sequence effect on velocity and to capture the effects of several beta bursts within each window, thereby increasing potential effect sizes. By considering long time periods, this approach was also relatively robust to systematic fluctuations in beta activity and burst probability within one rotatory movement cycle (lasting ~50 ms) that have been reported in the gait cycle (Fischer et al., 2018) and during repetitive index finger to thumb taps (Androulidakis et al., 2008).

## 3 Results

### 3.1 Behavioural results of change in movement over time

After artefact removal, the analysed movement time was 89.13 ± 1.5 s (29.7 ± 0.4 s for each of the three movement blocks) and rest time 91.8 ± 4.4 s per subject (drawn from the three corresponding rest blocks). Patients performed rotatory movements with a mean frequency of 1.7 ± 0.2 Hz and a mean speed of 240.7 ± 22.4 degrees/s. Three movement blocks were performed. For analysis, each block was subdivided in three equally long time windows (9.9 ± 0.1 s per time window) to assess the change of behaviour and oscillatory activity over time. A linear mixed-effects regression model (with averaged movement velocity in a given time window as the dependent variable, time windows as independent variables, random intercepts for different subjects and fixed slope between time window and movement velocity) demonstrated a significant relationship between averaged velocity and time window within blocks as well as across blocks (*n* = 12, estimate of fixed slope across blocks = −0.27, *P* = .006; estimate of fixed slope across windows within blocks = −0.24, *P* = .01, BIC = 100.87) without significant interaction between time windows and blocks (*P* = .2). Although the averaged velocity decreased from the first to the last block, the change in velocity within 10 s was similar both across windows within a movement block and across movement blocks (block: *P* = .1; window: *P* = .7). Summing up, these results confirm a decrease in velocity (sequence effect) over 30 s on a group level, with the slope of this velocity decrease being relatively uniform over time. An example of the performance of a single block is shown in Fig. 1B and the group data across and within blocks are summarised in Fig. 1C.

### 3.2 Power spectra at rest and during movement

**Supplementary Fig. 2:**
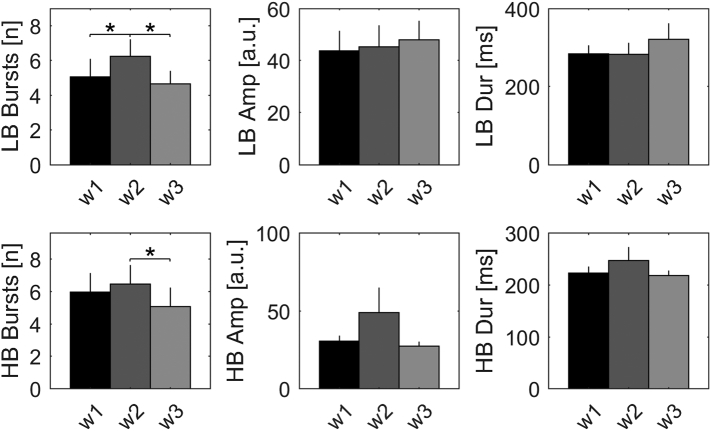
Burst properties within movement blocks.

When averaging beta band power separately for rest and movement periods, both low (13–20 Hz) and high beta (20–35 Hz) power were suppressed during movement (low beta: rest = 1.42 ± 0.2% total power, mov = 1.16 ± 0.2%, P_movrest_ = 0.02; high beta: rest = 0.86 ± 0.2%, mov = 0.55 ± 0.15%, P_movrest_ = 0.005; Fig. 2A, B). After z-scoring the low and high beta bands separately over the entire recording, mean power was assessed across the three windows comprising the blocks of movement. In the low beta band, the power suppression was significant in the first and last window but did not reach significance in the second window (rest = 0.1 ± 0.04; W1/W2/W3: mov = −0.155 ± 0.08/−0.0465 ± 0.07/−0.09 ± 0.06, P_movrest_ = 0.01/0.9/0.03; Fig. 2C and Supplementary Fig. 2). In the high beta band, the power suppression persisted across all time windows (rest = 0.17 ± 0.06; W1/W2/W3: mov = −0.17 ± 0.02/−0.13 ± 0.09/−0.22 ± 0.07, P_movrest_ = 0.01/0.02/0.003; Fig. 2D).

### 3.3 Movement-related modulation of beta burst properties

Beta burst properties at rest and during movement are summarised in Fig. 2E and F. Low beta bursts were present in all subjects during movement for 18.7 ± 3% of the total movement time (50 ± 7 bursts per subject, 16 ± 8 per block, 5 ± 0.8 per window) while high beta bursts were detected in 9/12 subjects during movement for 13 ± 3% of the total movement time (55.9 ± 7 bursts per subject, 17.5 ± 11 per block, 5.8 ± 1.1 per window). The 3/12 subjects without high beta bursting during movement showed no significant differences in peak velocity (HBeta Bursts Negative: 1.5 ± 0.18 a.u., HBeta Bursts Positive: 1.8 ± 0.21 a.u., *P* = .11) or change in velocity over time (HBeta Bursts Negative: −0.2 ± 0.15 a.u., HBeta Bursts Positive:–0.1 ± 0.26 a.u., *P* = .45). In both beta sub-bands, bursts occurred less frequently (low beta burst rate: rest = 0.7 ± 0.1 Hz, mov = 0.6 ± 0.3 Hz, *P* = .05; high beta burst rate: rest = 0.9 ± 0.1 Hz, mov = 0.5 ± 0.5 Hz, *P* = .008) and were shorter in duration (low beta burst dur: rest = 340 ± 62 ms, mov = 287 ± 86 ms, *P* = .04; high beta burst dur: rest = 278 ± 35 ms mov = 230 ± 51 ms, *P* = .02) during movement than at rest. In the low beta band, burst amplitude was also significantly smaller during movement when compared to rest (low beta burst amplitude: rest = 68 ± 22 a.u., mov = 43 ± 21 a.u., *P* = .02). This was not the case for high beta bursting (high beta burst amplitude: rest = 49 ± 16 a.u., mov = 38 ± 27 a.u., *P* = .25). The proportion of short bursts (≤300 ms) was increased during movement compared to an increased proportion of longer bursts (≥600 ms) at rest (see Fig. 2E and Supplementary Table 1).

Above we confirmed a decrease in velocity (sequence effect) over 30 s, but showed that the slope of this decrease in velocity was relatively constant over time. Similarly, mean burst amplitude, duration and rate, did not vary systematically with block order or time window indicating that beta bursting during movement was also relatively unaffected by the time within a given subject. This was shown by a linear mixed-effects regression model with a random intercept and fixed slope between subjects and mean burst duration, amplitude or rate as the dependent variable and movement block and time window within block as independent variables, separately for each beta frequency sub-band. The burst rate and the mean burst amplitude and duration were significant predictors of the averaged beta power within time windows (low beta: P_Rate_ < 0.001; P_Amp_ < 0.001; P_Dur_ = 0.01; high beta: P_Rate_ < 0.001; P_Amp_ < 0.001; P_Dur_ = 0.025). Burst rate was the most important predictor (low beta: coefficients estimate for burst rate = 0.63, coefficients estimate for z-scored burst amplitude = 0.003, coefficients estimate for burst duration = 0.0004; high beta: coefficients estimate for burst rate = 0.37, coefficients estimate for z-scored burst amplitude = 0.003, coefficients estimate for burst duration = 0.0004).

### 3.4 Correlation between decrement in movement velocity and beta bursting

**Fig. 3:**
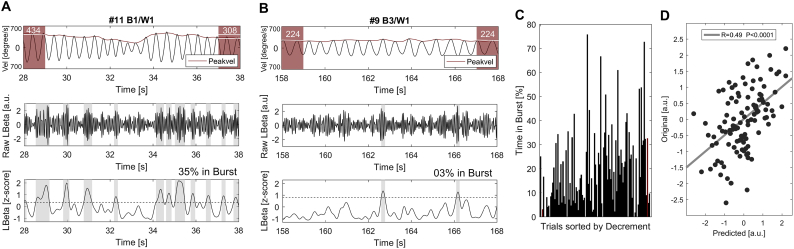
Correlation between low beta bursting and decrease in peak velocity. (A) Example of the first 10 s (w1) in the first movement block (b1) from case 11 is shown. The peak velocity trace (red line, first row) is an interpolation of peaks of movement velocity (black line, first row) to give a continuous estimation. Note that in off-line analysis, the change in peak velocity within a given 10 s time window was assessed by averaging peak velocity over the first and last second (red shaded area) and subtracting the two values (mean peak velocity in first second – mean peak velocity in last second, here: 434–308 degrees/s.; see white lines). In the second row is shown the corresponding raw trace of the local field potential recording, filtered around the low beta band (13–20 Hz). The third row demonstrates the fluctuations of the low beta amplitude after wavelet transform. The burst threshold of this subject is shown as dotted grey line and bursting activity (with a minimum duration of 100 ms) is highlighted in light grey. Here, 34% of time is spent in low beta bursting, which is associated with an overall decrement across the whole 10s window. (B) Same but now for a window with sparse bursting throughout (3%) and maintained peak velocity. Shown are the first 10 s (w1) in the third movement bloc (b3) from case 9. (C) In red are labelled the two example cases when trials from all subjects (*n* = 108) are sorted by the change of peak velocity over 10 s of continuous movement. (D) Correlation plot showing the decrease in peak velocity over 10 s from all subjects as predicted by our statistical model on the x-axis and the original decrease in peak velocity over 10 s on the y-axis. Change in peak velocity over 10 s could be significantly (*r* = 0.498, *P* < .0001) predicted by time spent in low beta bursts, explaining 25% of the variance in peak velocity change across patients. Shown values are normalized. (For interpretation of the references to colour in this figure legend, the reader is referred to the web version of this article.)

**Fig. 4:**
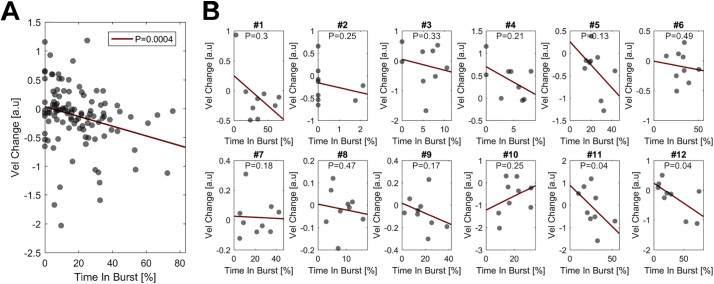
Correlation between change in velocity and time in burst within and across subjects. (A) Shown are all measurements of change in velocity and time spent in low beta bursts within and across subjects as independent values. This shows a highly significant, negative correlation (R = –0.31, *P* = .004). (B) Shown are individual scatter plots of the change in velocity over ~10 s (y-axis, negative values indicate a decrease in velocity over time) and the percentage time spent in low beta bursting over the same ~10 s (x-axis). In 10/12 cases, there was a negative correlation (fitted regression line shown in red) between change in velocity and time spent in low beta bursts that was individually significant in 2/10 cases. In case 10 there was a positive correlation and in case 2 beta bursting was detected only in 2/9 assessed time windows which limits interpretability. Across patients, the regression slope between change in velocity and low beta bursting was −2.2 ± 0.96 (mean ± SE). (For interpretation of the references to colour in this figure legend, the reader is referred to the web version of this article.)

Linear mixed effects regression models were used to assess the relationship between change in velocity and mean power or bursting in one or other of the beta sub-bands. Separate models were applied to data averaged within 3 (~10s) or 6 (~5 s) time windows. Model results are summarised in Table 2 and an example movement trace with corresponding low beta band activity is shown in Fig. 3A and B. More time spent in low beta bursts was associated with a drop in movement velocity in the respective window both when averaging within 3 (~10 s duration) and 6 (~5 s duration) time windows. This was shown by the negative slope estimates in the significant linear mixed effects regression models that assessed the relationship between change in velocity and bursting in the low beta sub-band (Table 2) and can be visually perceived when trials are sorted across subjects by the decrease in peak velocity over time (Fig. 3C). Considering only percentage time in low beta bursts as predictor explained 25% of the variance in velocity change in subjects (*r* = 0.498, *r*^2^ = 0.248, *P* < .0001). Fig. 3D shows the correlation between the decrease in peak velocity over 10 s from all subjects as predicted by our statistical model and the observed decrease in peak velocity over 10 s. It was consistent with the results of linear regression applied to each subject separately, which showed that the regression slope was −2.2 ± 0.96 (mean ± SE) when averaged across patients. The regression slope was negative in 10 out of the 12 subjects (Fig. 4 for individual scatter plots), with a significant negative correlation in 2/12 subjects. When considering all measurements of change in velocity and time in burst within and across subjects as independent, the negative correlation was highly significant (R = –0.31, *P* = .004), see Fig. 4A. Simple models with percentage time in higher frequency beta bursts, averaged low beta power or decrease in low beta power as fixed effects were not significant. When averaged low beta power, change in low beta power or percentage time in higher frequency beta bursts were considered as a predictor in addition to percentage time in low beta bursts, the model performance deteriorated, although percentage time in low beta bursts remained a significant predictor. Likewise, the model prediction remained significant but deteriorated in performance when increasing the beta band width (13–22 Hz, 13–24 Hz, 13–26 Hz, 13–28 Hz), changing the beta burst threshold (55th, 65th, 85th percentile) or normalizing to the rest period prior to the first movement block instead of the entire recording time (Supplementary Table 2). All significant models remained so after transforming model inputs to a normal distribution. Summing up, time spent in low, but not high, beta bursts correlated with a decrease in velocity, and this correlation was regardless of the mean beta power or change in beta power in the same windows.

**Table 2:** Change in movement velocity as dependent variable in all tested models.

Predictor		Estimate of slope	t-value	*P*-value	BIC
Relative time in low beta bursts	3w	−0.93	−2.9	**.005**	182.9
	6w	−0.39	−2.4	**.02**	243.3
Averaged low beta power	3w	−0.37	−1,7	.09	184.9
	6w	−0.13	−1.5	.12	246.6
Change in low beta power	3w	−0.03	−0.32	.7	190.85
	6w	0.003	0.07	.94	248.9
Relative time in high beta bursts	3w	−0.019	−0.04	.96	190.9
	6w	−0.006	−0.02	.98	248.9
Relative time in low beta bursts + Averaged low beta power	3w	−1.35	−1.25	.08	191.77
		0.26	0.622	.53	
Relative time in low beta bursts + Change in low beta power	6w	−0.94	−2.8	**.005**	192.36
		0.005	0.05	.53	
Relative time in low beta bursts + Relative time in high beta bursts	3w	−1.17	−3.3	**.001**	190.1
		0.72	1.6	.11	
	6w	−0.5	−2.8	.004	251.6
		0.35	1.6	.1	

3w = three 10s windows; 6w = six 5 s windows. BIC = Bayesian information criterion; *P* values in bold are significant after FDR.

## 4 Discussion

We investigated the temporal dynamics of subthalamic beta activity during continuously performed alternating movements in PD patients withdrawn from their regular medication. We demonstrate that distinct bursts of low beta activity that cross resting beta thresholds occur during motor performance even when patients were instructed to carry out movements as fast as possible. Movement velocity progressively decreased within blocks of continued movement, reflecting the sequence effect, which is a core feature of bradykinesia in PD. The amount of time spent above the low beta burst threshold as defined at rest was a significant predictor of the decrease in movement velocity, whereas averaged low beta power or change in low beta power were not. The relation to the decrease in velocity was specific for low beta bursts and not present for high beta bursts. Time spent in low beta bursts explained 25% of the variance in peak velocity change.

### 4.1 Beta bursts during movement

Neurons in the motor network are known to synchronize in the beta band (13–35 Hz) within and across brain areas involved in motor processing (Oswal et al., 2016; Neumann et al., 2015; Litvak et al., 2011b; Engel & Fries, 2010; Baker et al., 1997; Deffains et al., 2016; Khanna & Carmena, 2017; Murthy & Fetz, 1996; Lipski et al., 2017; Kühn et al., 2005). The beta band synchronization tends to occur transiently, in beta bursts (Tinkhauser et al., 2017b; Leventhal et al., 2012; Shin et al., 2017; Lofredi et al., 2018a; Sherman et al., 2016; Feingold et al., 2015), and most likely reflects a state of low likelihood for imminent change in the sensorimotor set (Engel & Fries, 2010; Gilbertson, 2005). The probability that beta bursts are longer and higher in amplitude is increased in untreated PD patients compared to patients treated with levodopa (Tinkhauser et al., 2017b) or DBS (Tinkhauser et al., 2017a). As such, it is presumed that beta bursting is exaggerated in untreated PD (Deffains et al., 2018), and that this is related to motor impairment. The latter is supported by the correlation between the shift to longer, larger bursts at rest and bradykinesia-rigidity scores (Tinkhauser et al., 2017a; Tinkhauser et al., 2017b). Indeed, even when patients' motor state is normalized as far as possible through treatment with levodopa, the presence of beta bursts in a time-limited window before movement onset still reduces the peak velocity of isolated ballistic movements with the effect being further amplified by the amplitude of the burst (Torrecillos et al., 2018). However, thus far the characteristics and correlates of beta bursts in PD have not been described during the continuous repetitive movements used to assess bradykinesia. One recent study demonstrated that although beta power was suppressed during continuously repeated movements, this suppression progressively diminished over time in tandem with a progressive decrement in the frequency and amplitude of movements (Steiner et al., 2017). However, this study did not consider the moment-to-moment dynamics of the beta power. We were able to confirm lower degrees of power during movement. Critically though, we showed that beta bursts still arose despite the overall beta suppression during movement. Bursts occurring during continuous movements were decreased in duration, amplitude and rate when compared to rest, despite being defined in the same way during movement as at rest. Nevertheless, the time spent in low beta bursts correlated with the decrease of movement velocity in PD patients performing repetitive movements, and this correlation was not simply explained by averaged beta power or change in beta power. This is in line with prior studies highlighting the rate of cortical beta bursts as a consistent predictor of behaviour in healthy animals (Shin et al., 2017; Sherman et al., 2016).

### 4.2 Implications for adaptive DBS

Currently, DBS paradigms continuously apply high-frequency stimulation, thereby suppressing subthalamic low beta activity (Oswal et al., 2016). Because the latter correlates with improvement of motor symptoms, subthalamic beta activity has emerged as a local feedback parameter for adaptive DBS (Little et al., 2013). Acutely triggering DBS only when a threshold of beta activity defined at rest is crossed has proven to be similar or even superior to continuous DBS in controlled conditions (Little et al., 2016a; Little et al., 2013; Little et al., 2016b). However, it has been unclear how adaptive stimulation delivered at a threshold defined at rest would be able to prevent bradykinesia when average beta power drops below resting levels during movement (Johnson et al., 2016). Our results reveal that beta burst events, although diminished in probability, amplitude and duration, can still surpass the threshold defined at rest and the time spent in such bursting is linked to the sequence effect of bradykinesia. The situation may be different during briefer movements. Here not all movements may necessarily be affected by beta bursts (Torrecillos et al., 2018). It is tempting to speculate that the distribution of movement velocities that includes normal speeds in patients with PD (Mazzoni et al., 2007) reflects the probabilistic nature of beta bursting during voluntary movement.

### 4.3 Limitations of the study

Having discussed the significance of our findings, it is prudent to address the limitations of this study. First, the correlation between low beta bursting and the sequence effect during repetitive movements may have been underestimated because intracerebral recordings were performed in the days following DBS surgery. This period is associated with a reduction in subthalamic beta activity and a temporary amelioration of parkinsonian symptoms (Chen et al., 2006; Mann et al., 2009). Second, studies in healthy control subjects performing repetitive finger tapping (Rodrigues et al., 2009; Ling et al., 2012) suggest that physiological fatigue also contributes to a progressive reduction in movement velocity. If we assume that beta bursts are associated with pathological and not physiological slowing, then overlapping fatigue will also tend to underestimate the contribution of beta bursts to the sequence effect. Third, we analysed the data from all studied subjects, but it should be noted that four subjects were classified as tremor dominant (see Table 1). This might have led to a weakening of the association between low beta bursting and the sequence effect across the group, although our correlative approach should have ameliorated this problem. Fourth, our findings are correlative, and by themselves, can only suggest and not prove that beta bursts may causally contribute to the sequence effect of bradykinesia. Fifth, episodes of manual freezing as defined as periods of zero velocity lasting one second or more were only observed in 3 instances in 2 subjects. These were too few to analyse, although we note that beta bursts have recently been associated with gait freezing (Syrkin-Nikolau et al., 2017). Finally, although not a confound, it is worth highlighting that the relevance of beta bursts and their features may not necessarily be the same during rest and during movement. Thus there may be time-limited periods of vulnerability during movement (Torrecillos et al., 2018) and other tasks (Khanna and Carmena, 2017) when even brief bursts are associated with a disturbance of function.

## 5 Conclusion

Here we show that subthalamic beta bursts can be detected during continuous movements in the dopamine-depleted state of PD patients. Critically, we reveal a spectrally specific relationship between the progressive decrement of movement velocity and the amount of bursting activity in the low beta band in the STN. Although the duration of beta bursts has been considered closely linked to motor impairment at rest, the present and other findings suggest that even briefer bursts can be deleterious when they occur during movement (Torrecillos et al., 2018; Sherman et al., 2016; Khanna and Carmena, 2017; Chen et al., 2006). These findings contribute to the understanding of the sequence effect in bradykinesia as a motor symptom that is specific for Parkinson's disease. Moreover, they have a direct bearing on adaptive DBS paradigms, and explain how a stimulation trigger defined at rest can still be effective during voluntary movement despite the coincident suppression of mean beta activity.

Supplementary material 1 Image 1

Supplementary material 2 Image 2
