# Supplementary material for: Beta bursts during continuous movements accompany the velocity decrement in Parkinson's disease patients
Source: Neurobiol Dis. 2019 Jul;127:462–71. doi: 10.1016/j.nbd.2019.03.013 (PMC6520224; doi:10.1016/j.nbd.2019.03.013)
Supplement: Supplementary file 1 — Supplementary material 1 [file mmc1.docx]

**Supplementary Table 1:**

| Low Beta Bursts | | | |
| --- | --- | --- | --- |
| Duration | Rest [% of all] | Movement [% of all] | P-value |
| > 100 - 200 ms | 46.3±2.4 | 61.82±5.6 | 0.01 |
| > 200-300 ms | 23.84±2.3 | 16.42±2.3 | 0.02 |
| > 400-500 ms | 10.55±0.54 | 7.39±1.4 | 0.03 |
| > 500-600 ms | 5.96±1.0 | 5.39±1.3 | 0.39 |
| > 600 ms | 13.37±2.1 | 8.96±2.0 | 0.04 |
| High Beta Bursts | | | |
| Duration | Rest [% of all] | Movement [% of all] | P-value |
| > 100 - 200 ms | 61.62± 1.6 | 74.01± 4.4 | 0.03 |
| > 200-300 ms | 17.23±1.4 | 13.44±1.8 | 0.03 |
| > 400-500 ms | 7.99±1.1 | 5.97±1.4 | 0.11 |
| > 500-600 ms | 5.16±0.7 | 2.75±0.9 | 0.07 |
| > 600 ms | 7.98±1.7 | 3.81±1.1 | 0.03 |
