# Supplementary material for: Beta bursts during continuous movements accompany the velocity decrement in Parkinson's disease patients
Source: Neurobiol Dis. 2019 Jul;127:462–71. doi: 10.1016/j.nbd.2019.03.013 (PMC6520224; doi:10.1016/j.nbd.2019.03.013)
Supplement: Supplementary file 2 — Supplementary material 2 [file mmc2.docx]

**Supp. Table 2: Change in movement velocity as dependent variable in all tested models**

| Predictor : Relative time in low beta bursts | Estimate of slope | t-value | P-value | BIC |
| --- | --- | --- | --- | --- |
| Normalised over entire trial |  | | | |
| Burst threshold : 75^th^ percentile |  | | | |
| - **Low Beta = 13-20 Hz** - Low Beta = 13-22 Hz - Low Beta= 13-24 Hz - Low Beta = 13-26 Hz - Low Beta = 13-28 Hz - Low Beta = 13-30 Hz | **-0.93**  -0.99  -0.97  -0.91  -0.87  -0.74 | **-2.9**  -2.7  -2.5  -2.3  -2.1  -1.7 | **.005**  .007 .01 .02  .04 .08 | **182.9**  183.7 184.6 185.7 186.7 187.9 |
| Low Beta = 13-20 Hz |  | | | |
| - Burst threshold : 55^th^ percentile - Burst threshold : 65^th^ percentile - **Burst threshold: 75^th^ percentile** - Burst threshold : 85^th^ percentile - Burst threshold : 95^th^ percentile | -0.64  -0.7  **-0.93**  -1.12  -1.8 | -2.4  -2.5  **-2.9**  -2.6  -1.8 | .02  .01  **.005**  .01  .07 | 185.2 184.9  **182.9**  184.4 187.8 |
| Normalised to pre-movement time |  | | | |
| - Low Beta (13-20Hz) + Burst Threshold 75^th^ percentile | -0.99 | -2.8 | .006 | 183.4 |

BIC = Bayesian information criterion; Underlined is the model as reported in the main results.
